# Sex-specific regulation of chemokine Cxcl5/6 controls neutrophil recruitment and tissue injury in acute inflammatory states

**DOI:** 10.1186/s13293-015-0047-5

**Published:** 2015-11-26

**Authors:** Shimona Madalli, Martina Beyrau, James Whiteford, Johan Duchene, Inderpal Singh Nandhra, Nimesh S. A. Patel, Madhur P. Motwani, Derek W. Gilroy, Christoph Thiemermann, Sussan Nourshargh, Ramona S. Scotland

**Affiliations:** Centre for Microvascular Research, London, EC1M 6BQ UK; Centre for Translational Medicine and Therapeutics, William Harvey Research Institute, Barts and The London Medical School, Queen Mary University of London, London, EC1M 6BQ UK; Department of Cardiovascular Research, Max Delbrück Center for Molecular Medicine (MDC), Berlin-Buch, Germany; Department of Medicine, Rayne Institute, University College London, London, WC1 6JJ UK

**Keywords:** Ischemia/reperfusion, Leukocyte, Bone marrow, Gender

## Abstract

**Background:**

Tissue infiltration by neutrophils during acute inflammatory states causes substantial tissue injury. While the magnitude of tissue neutrophil accumulation in innate immune responses is profoundly greater in males than females, fundamental aspects of the molecular mechanisms underlying these sex differences remain largely unknown.

**Methods:**

We investigated sex differences in neutrophil stimulation and recruitment in ischemia/reperfusion (I/R; mesenteric or renal) or carrageenan pleurisy in rats or mice, as well as skin injury in human volunteers. The induction of potent chemoattractive mediators (chemokines) and neutrophil adhesion molecules were measured by real-time PCR, flow cytometry, and protein assays.

**Results:**

Mesenteric I/R in age-matched Wistar rats resulted in substantially more neutrophil accumulation and tissue injury at 2 h reperfusion in males than females. Using intravital microscopy, we show that the immediate (<30 min) neutrophil response to I/R is similar in males and females but that prolonged neutrophil recruitment occurs in males at sites local and distal to inflammatory insult partly due to an increase in circulating neutrophil populations with elevated surface expression of adhesion molecules. Sex differences in neutrophil kinetics were correlated with sustained induction of chemokine Cxcl5 in the tissue, circulation, and bone marrow of males but not females. Furthermore, blockade of Cxcl5 in males prior to ischemia resulted in neutrophil responses that were similar in magnitude to those in females. Conversely, administration of Cxcl5 to males in the absence of I/R was sufficient to increase levels of systemic neutrophils. Cxcl5 treatment of bone marrow neutrophils in vitro caused substantial induction of neutrophil-mobilizing cytokine granulocyte colony-stimulating factor (GCSF) and expression of β2 integrin that accounts for sexual dimorphism in circulating neutrophil populations in I/R. Moreover, male Cxcl5-stimulated bone marrow neutrophils had an increased capacity to adhere to β2 integrin ligand ICAM-1, implicating a greater sensitivity of male leukocytes to Cxcl5-mediated activation. Differential induction of Cxcl5 (human CXCL6) between the sexes was also evident in murine renal I/R, rat pleurisy, and human skin blisters and correlated with the magnitude of neutrophil accumulation in tissues.

**Conclusions:**

Our study reveals that sex-specific induction of chemokine Cxcl5/CXCL6 contributes to sexual dimorphism in neutrophil recruitment in diverse acute inflammatory responses partly due to increased stimulation and trafficking of bone marrow neutrophils in males.

**Electronic supplementary material:**

The online version of this article (doi:10.1186/s13293-015-0047-5) contains supplementary material, which is available to authorized users.

## Background

Neutrophils are essential for early innate immune responses in infection and sterile injury such as ischemia/reperfusion (I/R). However, due to their high destructive potential, regulation of accumulation of neutrophils in tissues is critical for limiting tissue injury and loss of function in acute inflammatory conditions (for review, see [1]).

While it is established that women are more susceptible to chronic inflammatory autoimmune diseases (for review, see [2]), profound sex differences also exist in severity and mortality from disorders that are characterized by prolonged or excessive innate immune responses (for review, see [3]). For example, the severity and incidence of I/R-induced organ failure and acute respiratory distress syndrome (ARDS) are substantially higher in men compared to those in women (for reviews, see [4–6]). While differential lifestyle/environmental factors between men and women influence outcome of these conditions, sex differences are also evident in experimental mammalian models of disease, intimating that this sex bias is an inherent feature in males. Elucidation of the male and female patterns of innate immune responses has important implications for understanding disease progression in men and women and therefore appropriate targeting of anti-inflammatory therapies [7]. We have previously demonstrated that acute exposure to bacteria and other irritants in male mice and rats result in increased severity of inflammation compared to females, associated with enhanced recruitment of neutrophils into tissues [8]. Similarly, other studies indicate increased neutrophil accumulation following cytokine treatment or I/R in males [9, 10]. However, many fundamental aspects of this differential regulation of neutrophils remain unclear, and thus, attempts to exploit such sex differences therapeutically have not yet been successful. For example, it remains unclear whether neutrophil responses in females involve distinct molecular pathways, or whether female responses are delayed or resolve earlier than in males. Thus, to understand the regulatory mechanisms that underlie differential neutrophil responses between the sexes, we have investigated the temporal regulation and molecular pathways of neutrophil recruitment in males compared to those in females.

Trafficking of neutrophils from the circulation into tissues is coordinated by a network of chemokines, particularly those harboring the ELR (Glu-Leu-Arg) motif (e.g., Cxcl1, Cxcl2) [11], which establish chemotactic gradients between blood and tissue as well as within tissues [12]. Neutrophils are recruited via the leukocyte adhesion cascade through multiple steps (e.g. rolling adhesion and emigration), mediated by adhesion molecules including selectins and integrins [13]. The bone marrow is considered to be the primary site of neutrophil production and a store of neutrophils that can be rapidly mobilized to increase the number of circulating neutrophils available for recruitment at sites of inflammatory insult. Therefore, regulation of neutrophil release from the bone marrow is a key step that dictates the magnitude and duration of neutrophil recruitment. Similar to recruitment from the blood into the tissues, trafficking of neutrophils out of the bone marrow into the circulation is also directed by ELR+ chemokines. The actions of ELR+ chemokines in bone marrow mobilization are a combined effect of direct interaction with neutrophil Cxcr2 receptors together with the negative regulation of the predominant bone marrow neutrophil retention pathway, i.e., neutrophil Cxcr4 and its ligand Cxcl12 (for review, see [14]).

We hypothesized that differential regulation of the chemokine network underlies sex differences in the magnitude of neutrophil recruitment. The aim of our study was to investigate sexual dimorphism in neutrophil kinetics and identify the specific chemokines responsible for bringing about these effects. We found that the onset of neutrophil infiltration into tissues was similar between the sexes but that the levels continued to increase with time in males due to substantial increases in circulating neutrophils. The predominant endogenous chemotactic mediator generated by I/R in males was ELR+ chemokine Cxcl5 (human CXCL6 [15]), which had a unique temporal profile compared to other chemokines that accounts for sustained neutrophil stimulation, mobilization, and recruitment during reperfusion. The greater induction of Cxcl5 in males was associated with more neutrophilia and consequently a higher magnitude of neutrophil recruitment and tissue injury than that in females. Therefore, these studies provide the first evidence for sex-specific regulation of Cxcl5/6 in acute inflammatory states.

## Methods

### Animals

Experiments were conducted on age-matched male and female Wistar rats (250–300 g; 8–10 weeks, Charles River) or C57BL6 mice (20–25 g; 8–10 weeks, Charles River) and approved by Animals Scientific Procedures Act (UK). For intravital microscopy, rats were kept on a restricted diet for 12 h to reduce intestinal motility during imaging.

### Intravital microscopy

Anaesthetized rats (pentobarbitone 60 mg/kg, ip) were prepared for intravital microscopy (IVM), as previously described [16]. Rats were placed on a heated (37 °C) viewing stage, and a loop of intestine was exposed to visualize the mesenteric microcirculation. The mesentery was superfused with Tyrode’s solution (5 % CO_2_, pH 7.4). Images were recorded using Pinnacle Studio software (v.9). In each animal, a single un-branched postcapillary venule (diameter 25–40 μm, length >400 μm) was selected for the study. The following parameters were measured: leukocyte flux, rolling velocity, adhesion, and emigration. Rolling leukocytes were observed as cells moving visibly slower than red blood cells and were measured by counting the number of rolling leukocytes passing a fixed reference point (flux) on the vessel segment over a 2-min period. Leukocyte rolling velocity was determined from the time required for a randomly chosen leukocyte to roll across 200 μm. Rolling velocities of six leukocytes were averaged and expressed as micrometer per second. Adherent leukocytes were identified as cells that remained stationary within the vascular lumen for a period of at least 30 s and were counted in four consecutive 100-μm vessel segments. Leukocyte emigration from the postcapillary venule into the tissue was quantified by counting the number of cells up to 50, 50–100, and 100–150 μm away from the vessel wall in parallel with 100-μm vessel segments. Four readings were taken for each vessel and quantified on one side of the vessel wall. Red blood cell centreline velocity was measured in venules using an optical Doppler velocimeter (Microcirculation Research Institute, Texas A&M University, USA). Mean red blood cell velocity was calculated from centreline velocity/1.6, and venular shear rate was determined based on the Newtonian definition (8000 × mean red blood cell velocity / venular diameter).

In some experiments, male rats were treated with Cxcl5 (3 μg/kg, ip, R&D Systems) and IVM conducted at 2 h.

### Mesenteric ischemia/reperfusion

Rats from the same litter were alternately assigned to sham or I/R group, with experiments conducted on males and females on alternate days. Ischemia was induced by occluding the superior mesenteric artery (SMA). After 30 min of ischemia, reperfusion was permitted for up to 2 h and leukocyte dynamics were measured by IVM every 15 min. Sham-operated animals underwent identical surgical procedures without occlusion of the SMA. Blockade of endogenous Cxcl5 was achieved by 1-h pre-treatment with Cxcl5 monoclonal antibody (20 μg/kg, iv, R&D Systems).

### Histology of mesentery

The intestinal wall was removed and portions of mesentery were fixed (4 % paraformaldehyde, 5 min) and dried onto slides overnight. Tissues were immersed in absolute alcohol (5 min) and washed with distilled water prior to staining with hematoxylin and eosin.

### Determination of intestinal necrosis

Following reperfusion, a 4-cm segment of the exposed intestinal wall was purged, cut into 2–3 mm cubes and incubated with *p*-nitroblue tetrazolium dye (NBT, 0.5 mg/ml, 20 min, 37 °C). Necrotic unstained portions were separated from viable stained tissue and expressed as a percentage of total wet weight of the segment.

### Lung myeloperoxidase activity

Lung tissues were gently rinsed, homogenized in 0.5 % (*w*/*v*) hexadacyl trimethylammomium bromide and centrifuged (13,000*g*, 10 min, 4 °C). Peroxidase activity of the supernatant was measured as the rate of H_2_0_2_-dependent oxidation of 3,3′,5,5′-tetramethylbenzidine relative to purified myeloperoxidase (MPO), by optical density (620 nm). Tissue MPO levels were normalized to total protein content.

### Collection and preparation of leukocytes and mesenteric tissue

Blood leukocytes were collected into 0.5 M EDTA. Peritoneal leukocytes were collected by lavage (10 ml PBS/0.25 % BSA/2 mM EDTA). Bone marrow (BM) cells were isolated from the femur by flushing with ice-cold PBS (rats, 5 ml; mice, 1 ml) and passing through a 70-μm cell strainer. Plasma and supernatants were collected by centrifugation (300*g*, 5 min, 4 °C) and stored at −80 °C for protein analysis. For all cell samples, erythrocytes were lysed and leukocytes were either snap frozen for RNA analysis or prepared for flow cytometry. Mesenteric tissues were isolated by separating the mesentery from the intestinal wall, snap frozen, and stored at −80 °C for RNA analysis.

### Flow cytometry

Leukocytes were resuspended in PBS/1 % goat serum (2 × 10^6^ cells/ml). Flow cytometry was conducted on BD FACScalibur™ with data analyzed by FlowJo 7.6.1. Leukocytes were fixed and permeabilized (Leucoperm, AbD Serotec) and incubated with antibodies (30 min, 4 °C) to leukocyte subset markers, integrins, or L-selectin (in live cells), using respective isotype antibodies as controls and compensated as appropriate for multiple labeling. Surface integrin and L-selectin expression was calculated as fold expression compared to isotype (relative fluorescence intensity: RFI). For additional details about antibodies, see Additional file 1: Table S1. Neutrophil shape change was assessed by increased ability of RP1+ leukocytes to scatter light in flow cytometer and expressed as percentage of neutrophils exhibiting high forward scatter (FSC^hi^), as previously described [17, 18].

### Quantification of chemokines and cytokines

RNA was extracted from leukocyte pellets and mesenteric tissue (NucleoSpin, Macherey-Nagel), reverse transcribed (Mouse Moloney Leukaemia Virus reverse transcriptase) and 20 ng cDNA submitted to quantitative real-time PCR (Applied Biosystems 7900HT), and quantified using SYBR® green (for primer sequences, see Additional file 1: Table S2). RNA levels of target genes were assessed by threshold cycle number (Ct) and normalized to Ct of house-keeping gene for 18S and calculated as fold expression relative to the mean Ct value of the control group, using ∆∆Ct method [19]. Chemokines in plasma, cell-free BM washouts, or blister fluid were measured by enzyme-linked immunosorbent assay (ELISA) according to the manufacturer’s instructions (R&D Systems: Cxcl1, Cxcl5, CXCL6; eBioscience: Ccl2).

### In vitro stimulation of leukocytes

BM leukocytes (5 × 10^5^) in phenol red-free RPMI medium (10 % fetal calf serum, 20 mM L-glutamine, 1000 U/ml penicillin/streptomycin) were plated in 48-well plates and stimulated with Cxcl1 or Cxcl5 (10 ng/ml, 2 h, R&D Systems). Cells were collected using EDTA cell dissociation buffer (Invitrogen).

### Leukocyte adhesion assay

BM leukocytes (10^5^ cells) were prepared in serum-/phenol red-free RPMI medium and stimulated with Cxcl5 (100 ng/ml, 1 h, R&D systems) prior to plating in black opaque 96-well plates coated with BSA (2 % *w*/*v*) or ICAM1 (1 μg/well) for 40 min (37 °C). Plates were prepared by coating wells with protein overnight and treated with BSA (1 % *w*/*v*, 1 h) to inhibit non-specific interactions prior to addition of leukocytes. Non-adherent leukocytes were removed by washing with Ca^2+^/Mg^2+^-free PBS. Adherent leukocytes were labeled with calcein AM (5 μM, 1 h), quantified by spectrophotometry (absorbance 485 nm), and expressed as a percentage of absorbance of 10^5^ labeled cells.

### Carrageenan pleurisy

Pleurisy was induced in Wistar rats by injection of 0.15 ml of 1 % carrageenan (*w*/*v*) into the pleural cavity. Pleural leukocytes were collected at 3 h by lavage (1 ml PBS/0.3 % citrate (*w*/*v*). Edema was assessed by the weight of excess fluid recovered from the pleural cavity.

### Renal I/R

Male and female C57BL6 mice were anesthetized with ketamine/xylazine (100 mg/kg, 10 mg/kg, ip), and renal pedicles were occluded using microvascular clamps. After 30-min bilateral ischemia, the clamps were removed and the skin was sutured. Analgesic buprenorphine (0.1 mg/kg, s.c.) was administered, and mice were allowed to recover for 24 h prior to harvesting samples.

### Human skin blisters

Ethical approval was obtained from University College London Ethics Board. Blisters were elicited on the ventral aspect of the forearms of healthy volunteers (eight men, six women, aged 19–32 years) by applying 10 μl of 0.1 % Cantharone (Dormer Labs, Inc.). Volunteers did not take any medications for 2 weeks before commencement of the study and abstained from exercise, alcohol, and caffeine for at least 24 h prior to induction of blister. Blister fluid was collected at 24 h and leukocytes were prepared for cytometry, as previously described [20]. After exclusion of CD3+ lymphocytes, neutrophils were identified as CD16^hi^/HLA-DR^−^ and monocytes as CD14^hi^/HLA-DR^+^.

### Statistical analysis

Data are expressed as mean ± sem. Comparisons between two groups were made by two-tailed unpaired Student’s *t* test. For comparisons between multiple groups, a one-way ANOVA was performed followed by Bonferroni’s post-test. Comparisons between time-response curves were made using a two-way ANOVA, followed by Bonferroni’s post-test. Blister samples were analyzed by non-parametric Mann-Whitney test. Statistical analysis was performed using Prism 5.0 (GraphPad Software Inc.).

## Results

### Distinct temporal regulation of neutrophil recruitment in females protects against I/R injury

**Fig. 1:**
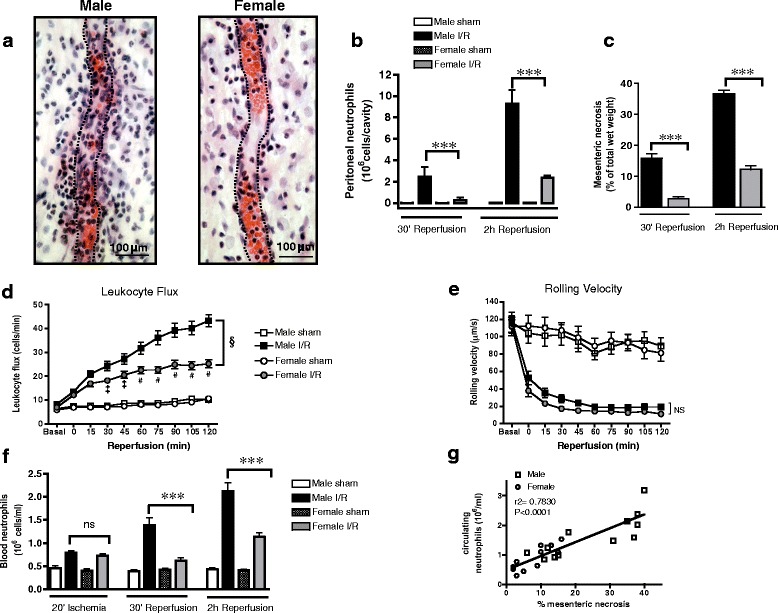
Distinct temporal regulation of neutrophil recruitment protects against I/R injury in females. Male and female rats were subjected to 30-min mesenteric ischemia followed by up to 2-h reperfusion. **a** Representative images of segments of male and female mesentery at 2 h of reperfusion, stained with hematoxylin and eosin, demonstrating fewer nucleated cells within and around female venules. *Dashed lines* approximately demarcate venule lumen. **b** Accumulation of peritoneal RP1+ neutrophils, measured by cytometry. **c** Proportion of intestinal necrosis, measured by nitroblue tetrazolium. *Sham n* = 5 rats/group, *I/R n* = 8 rats/group. **d**, **e** Leukocyte/vessel wall interactions throughout reperfusion in mesenteric venules, measured by intravital microscopy: **d** leukocyte flux and **e** leukocyte rolling velocity (*sham n* = 11 rats/group, *I/R n* = 18 rats/group). **f** Circulating RP1+ neutrophils, measured by flow cytometry. **g** Direct correlation between number of circulating neutrophils and extent of intestinal necrosis in both males and females, measured at 30-min and 2-h reperfusion. *Sham n* = 5 rats/group, *I/R n* = 8 rats/group. Data are presented as mean ± sem. ***P < 0.001 by one-way ANOVA. §*P* < 0.001 by two-way ANOVA, and ‡*P* < 0.05 or #*P* < 0.001 by Bonferroni’s post-test compared to male I/R. *ns* denotes *P* > 0.05

To understand whether sex differences exist in the temporal regulation of leukocytes in acute inflammatory responses, we subjected male and female rats to 30-min complete mesenteric ischemia followed by 2-h reperfusion. Histology of the mesenteric vasculature revealed substantially more cell infiltration at the end of reperfusion in male tissues than that in females (Fig. 1a). FACS analysis identified recruitment of RP1+ neutrophils into the peritoneal cavity in both sexes, but levels were significantly greater in males than those in females (Fig. 1b). In addition to increased neutrophil recruitment in males, substantial redness and edema was evident in the small intestine at the end of reperfusion in males but not females (Additional file 2: Figure S1A). Quantification of intestinal wall necrosis using NBT [21] confirmed that the extent of tissue injury was significantly more in males at both 30-min and 2-h reperfusion (Fig. 1c).

To directly examine the events that precede enhanced I/R-stimulated neutrophil sequestration in male tissues, we used intravital microscopy to visualize leukocyte/vessel wall interactions throughout the reperfusion period. The rate of leukocytes interacting with mesenteric venules (leukocyte flux) increased from the onset of reperfusion in both sexes, but the increase was less in females and plateaued at 1 h whereas leukocyte flux continued to increase with reperfusion time in males (Fig. 1d). Dampened leukocyte flux in females was also accompanied by fewer adherent leukocytes to the vessel lumen and subsequent emigration into the mesenteric tissue (Additional file 2: Figure S1B-C). These differences in leukocyte dynamics were not a consequence of differences in I/R-induced changes in hemodynamics (mean arterial blood pressure, red blood cell velocity, or wall shear rate) since no significant change from basal values was evident during reperfusion or between the sexes (Additional file 3: Figure S2A-C).

While leukocyte flux in the mesenteric vasculature was substantially more in males, the velocity of rolling leukocytes was similar in both sexes implicating a difference in the availability of leukocytes rather than in their capacity to interact with venules (Fig. 1e). Consistent with this, neutrophil adhesion molecule L-selectin expression (RFI) at 2-h reperfusion was 6.8 ± 0.93 and 5.9 ± 0.62 (*n* = 4, *P* > 0.05) in males and females, respectively. Therefore, we hypothesized that a key difference in the response to I/R in males and females involves the regulation of release of neutrophils from the bone marrow. Indeed, reperfusion stimulated a time-dependent surge in circulating neutrophils in males but had little effect on blood neutrophil populations in females (Fig. 1f). In contrast, the modest I/R-induced changes in circulating monocyte numbers were similar in both sexes, suggesting a selective differential regulation of neutrophil mobilization in males (Additional file 2: Figure S1D). Moreover, the levels of circulating neutrophils were directly correlated (*r*^2^ = 0.7830, Fig. 1g) with the extent of I/R-induced tissue necrosis in both sexes, supporting a causative link between mobilization of neutrophils and I/R injury.

### Enhanced bone marrow neutrophil stimulation in males

**Fig. 2:**
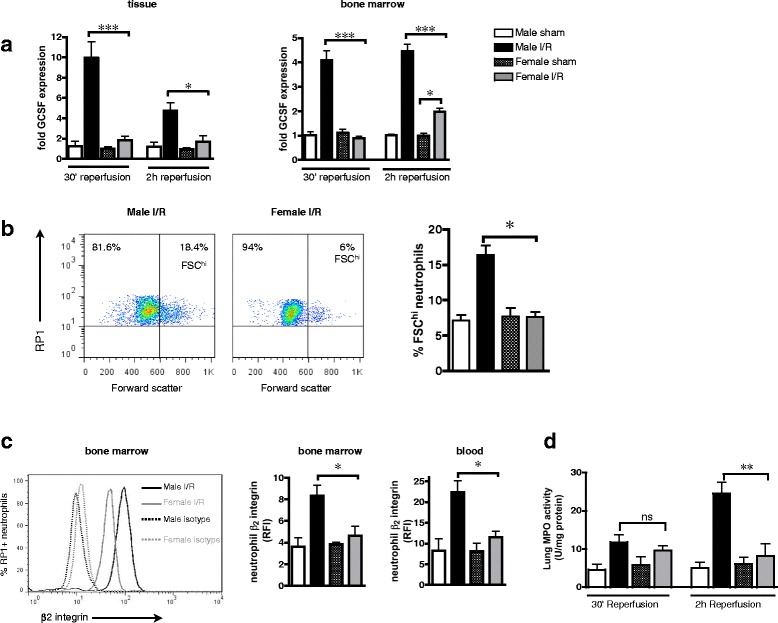
Increased bone marrow neutrophil stimulation in males. Male and female rats were subjected to 30-min mesenteric ischemia followed by up to 2-h reperfusion. **a** Expression of mesenteric tissue and bone marrow cell GCSF mRNA, normalized to levels of 18S and calculated as fold expression relative to mean value of male sham group. **b** Bone marrow neutrophil shape change measured by cytometry as the proportion of RP1+ cells with high forward scatter (FSC^hi^), at 2-h reperfusion. **c** Bone marrow and blood neutrophil β2 integrin expression at 2-h reperfusion, measured by cytometry and expressed as relative fluorescence intensity (RFI) compared to isotype control antibody. **d** Neutrophil accumulation in the lungs, assessed by tissue MPO activity. *Sham n* = 5 rats/group, *I/R n* = 8 rats/group. Data are presented as mean ± sem. *P < 0.05, **P < 0.01, ***P < 0.001, and ns *P* > 0.05 by one-way ANOVA

The finding that circulating neutrophil numbers are elevated during reperfusion indicates that I/R triggers bone marrow neutrophil stimulation and mobilization in males but not females. Acute granulopoeisis and neutrophil maturation/mobilization during infection are controlled by granulocyte colony stimulating factor (GCSF) [22]. Accordingly, I/R induced an early and sustained synthesis of GCSF by bone marrow cells and ischemic mesenteric tissue in males but not females (Fig. 2a). These results imply that increased GCSF levels in males stimulate enhanced bone marrow neutrophil motility compared to those in females during reperfusion. Indeed, we noted a significant increase in the size of individual bone marrow neutrophils in males at 2-h reperfusion, indicative of neutrophil shape change [17, 18] (Fig. 2b). Furthermore, surface integrin expression (β2: Fig. 2c; β1, α4, αL, αM: Additional file 4: Figure S3) on the bone marrow and circulating neutrophils at 2-h reperfusion was significantly elevated in males but remained unchanged in females. A possible functional consequence of increased circulating neutrophils with high integrin expression is that neutrophils can infiltrate other tissues remote to the site of inflammatory insult. In particular, secondary injury of the lung due to inappropriate neutrophil recruitment is a common consequence of I/R (for review, see [23]). Myeloperoxidase (MPO) activity in lung tissue, an index of neutrophil accumulation, remained unaltered in the lungs of female rats but was elevated fivefold in males by the end of reperfusion (Fig. 2d). Thus, these studies strongly indicate that more bone marrow neutrophils are mobilized in males during I/R and that neutrophils circulating during reperfusion exhibit a greater adhesive phenotype compared to females. Together, this differential regulation of neutrophils may protect against local and remote tissue injury in females.

### Sustained release of tissue-derived Cxcl5 mediates bone marrow neutrophil stimulation and I/R injury in males but not females

**Fig. 3:**
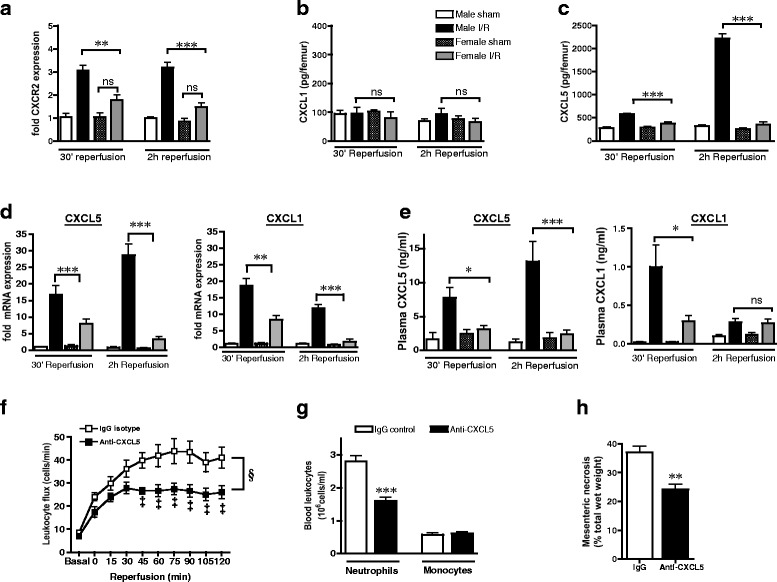
Sustained release of tissue-derived Cxcl5 throughout reperfusion in males but not females contributes to I/R injury. **a**–**e** Male and female rats were subjected to 30-min mesenteric ischemia followed by up to 2-h reperfusion. **a**–**c** Expression of bone marrow neutrophil mobilization pathways: **a** bone marrow cell Cxcr2 mRNA, **b** bone marrow total Cxcl1 protein, and **c** bone marrow total Cxcl5 protein. *Sham n* = 4 rats/group, *I/R n* = 6 rats/group. **d** Mesenteric tissue mRNA levels of Cxcl5 and Cxcl1. Levels of mRNA are normalized to 18S and calculated as fold expression relative to mean value of male sham group. *Sham n* = 3 rats/group, *I/R n* = 6 rats/group. **e** Plasma protein levels of Cxcl5 and Cxcl1, at 30-min and 2-h reperfusion. *Sham n* = 5 rats/group, *I/R n* = 7 rats/group. **f**–**h** Male rats were treated with either anti-Cxcl5 (20 μg/kg, iv) or control IgG (20 μg/kg, iv) 1 h prior to mesenteric ischemia (*n* = 5 rats/group). **f** Leukocyte/vessel wall interactions in mesenteric venules throughout reperfusion. **g** Circulating RP1+ neutrophils and CD68+ monocytes. **h** Mesenteric necrosis, measured by nitroblue tetrazolium at 2-h reperfusion. Data are presented as mean ± sem. *P < 0.05, **P < 0.01, ***P < 0.001, ns *P* > 0.05 by one-way ANOVA. §*P* < 0.0001 by two-way ANOVA and ‡*P* < 0.05 by Bonferroni’s post-test

While GCSF is essential for regulation of neutrophil number in the bone marrow, egress of mature neutrophils into the circulation in acute inflammatory states is partly dependent on engagement of neutrophil chemokine receptor Cxcr2 with ELR+ chemokines such as Cxcl1 [24]. In keeping with a central role of Cxcr2 for exit of neutrophils from the bone marrow, expression of Cxcr2 mRNA by bone marrow cells was elevated throughout reperfusion in males but not females (Fig. 3a). Chemokines Cxcl1 and Cxcl2 are considered to be the most important Cxcr2 ligands that promote neutrophil mobilization from bone marrow stores during acute infections. However, the predominant endogenous chemokines that are released during vascular injury, such as I/R, which regulate bone marrow neutrophil trafficking are not known. Surprisingly, protein levels of Cxcl1 within the bone marrow were not modulated by I/R in either sex (Fig. 3b) whereas Cxcr2 ligand Cxcl5 was upregulated by I/R and increased with reperfusion time in males, with no discernible change in Cxcl5 in females (Fig. 3c).

Pro-mobilization signals during infection typically arise from local tissue production of cytokines and chemokines at the site of infection. To verify the primary cellular source of I/R-induced chemokine production, we measured chemokine mRNA in mesenteric tissue at 30-min and 2-h reperfusion. In males, tissue Cxcl5 mRNA was rapidly induced upon reperfusion and increased with reperfusion time whereas induction of chemokines Cxcl1 and Ccl2 were greater at 30 min and had declined by 2 h (Fig. 3d and Additional file 5: Figure S4A). The profile of circulating levels of chemokine protein mirrored the profile of tissue mRNA levels (Fig. 3e and Additional file 4: Figure S4B), implicating the ischemic tissue as a principle source of these chemokines. Importantly, levels of both tissue and circulating chemokines were less in females with the greatest fold difference being release of Cxcl5 (Fig. 3d). Sex differences in chemokine levels were specific to ligands for Cxcr2 or Ccr2 since induction of chemokines Ccl3/MIP1α or Ccl5/RANTES (ligands for Ccr1+ and Ccr5+ leukocytes) was similar in both sexes (Additional file 5: Figure S4C-D). The unique profile of Cxcl5 synthesis and its differential induction between the sexes indicates that this particular chemokine may be a pivotal endogenous mediator of I/R-induced neutrophil responses and tissue injury. Neutralization of Cxcl5 in males reduced plasma Cxcl5 at 2-h reperfusion from 10.2 ± 2.43 to 2.0 ± 0.37 ng/ml (*P* < 0.05, *n* = 5) and dampened I/R-induced leukocyte dynamics (Fig. 3f, Additional file 5: Figure S4C-D) to similar levels as those seen in females (Fig. 1d). The effect of anti-Cxcl5 on leukocyte recruitment was partly due to a reduction in neutrophilia (Fig. 3g), which was also limited to a similar magnitude as that in females (Fig. 1f). Collectively, these studies demonstrate that augmented levels of Cxcl5 in males account for increased neutrophilia during I/R compared to females.

### Cxcl5 induces bone marrow neutrophil stimulation in males

**Fig. 4:**
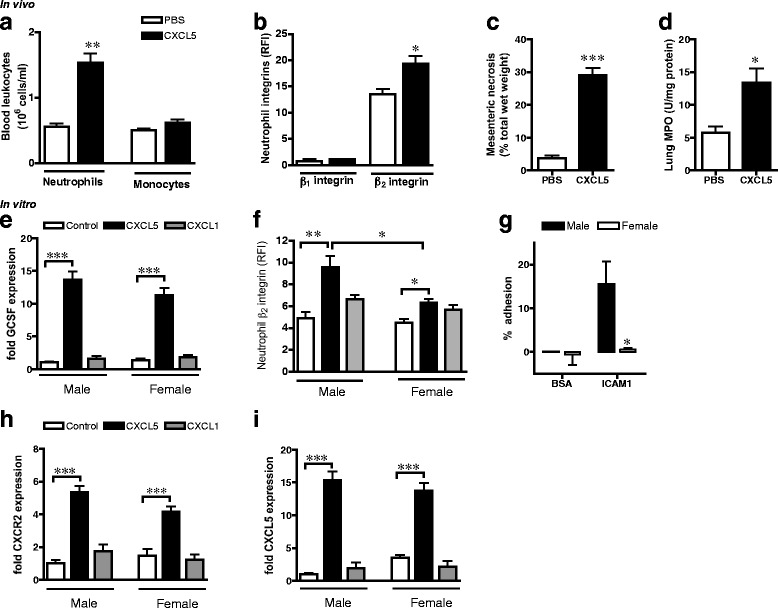
Cxcl5 induces neutrophil stimulation and mobilization pathways. **a**–**d** Male rats were administered 3 μg/kg Cxcl5 (ip, *n* = 5 rats) or sterile PBS (*n* = 3 rats) for 2 h. **a** Circulating RP1+ neutrophils and CD68+ monocytes. **b** Expression of β1 and β2 integrins on bone marrow RP1+ neutrophils. **c** Mesenteric necrosis, measured by nitroblue tetrazolium and **d** lung MPO levels. (**e**–**f**, **h**–**i**) Bone marrow cells (5 × 10^5^) were isolated from male and female rats (*n* = 4 rats/group) and treated with Cxcl5 (10 ng/ml) or Cxcl1 (10 ng/ml) for 2 h. **e** Bone marrow cell GCSF mRNA expression. **f** Expression of neutrophil β2 integrin, measured by cytometry. **g** Isolated male and female bone marrow cells (10^5^) were stimulated with Cxcl5 (100 ng/ml, 1 h) prior to incubation in 96-well plates for 40 min. Adhesion of cells to wells coated with BSA (2 %) or ICAM1 (1 μg/well), quantified using calcein-AM (*n* = 3 rats/group). **h**–**i** Bone marrow cell mRNA expression of **h** Cxcr2, **i** Cxcl5. Levels of mRNA are normalized to 18S and calculated as fold expression relative to mean value of male control group. Data are presented as mean ± sem. *P < 0.05, **P < 0.01, ***P < 0.001, ns *P* > 0.05 by one-way ANOVA

To recapitulate the impact of tissue-derived Cxcl5 on bone marrow neutrophil stimulation and mobilization, we administered Cxcl5 (3 μg/kg) locally into the peritoneal cavity of male rats and measured neutrophil populations at 2 h. This dose of Cxcl5 increased circulating levels at 2 h from 0.3 ± 0.13 to 7.7 ± 0.80 ng/ml (*P* < 0.001, *n* = 5), bone marrow levels from 0.1 ± 0.034 to 1.8 ± 0.39 ng/femur (*P* < 0.05, *n* = 5), and stimulated similar levels of leukocyte recruitment into mesentery (Additional file 6: Figure S5A-C) as I/R (Fig. 1d, Additional file 1: Figure S1B&C). Administration of Cxcl5, in the absence of ischemia, increased circulating neutrophils, but not monocytes, as well as neutrophil β2 integrin (Fig. 4a,b) and was sufficient to induce significant tissue injury at the site of administration and in the lungs (Fig. 4c,d).

To identify the molecular pathways in Cxcl5-induced bone marrow neutrophil stimulation/mobilization, rat bone marrow cells were treated with Cxcl5 (10 ng/ml, 2 h) in vitro. The proportion of RP1+ neutrophils isolated from femurs was similar (*P* > 0.05, *n* = 4) in both sexes (29.1 ± 0.59 and 29.1 ± 1.11 % in males and females, respectively). Cxcl5, but not Cxcl1, increased GCSF mRNA in both sexes (Fig. 4e), implicating that Cxcl5 can influence bone marrow neutrophil numbers and stimulation. Consistent with this notion, Cxcl5 promoted expression of β2 integrins in RP1+ neutrophils (Fig. 4f). Notably, this effect of Cxcl5 was significantly greater in male neutrophils compared to females, as confirmed by the increased ability of Cxcl5-stimulated male leukocytes to adhere to β2 integrin ligand ICAM1 (Fig. 4g). Cxcl5, but not equivalent amounts of Cxcl1, stimulated expression of Cxcr2 as well as Cxcl5 in leukocytes from both sexes (Fig. 4h,i). These studies confirm the hypothesis that elevated Cxcl5 activity in males is sufficient to directly increase bone marrow neutrophil stimulation and mobilization pathways.

### Differential regulation of Cxcl5 in males is a fundamental feature of I/R and other innate immune responses

**Fig. 5:**
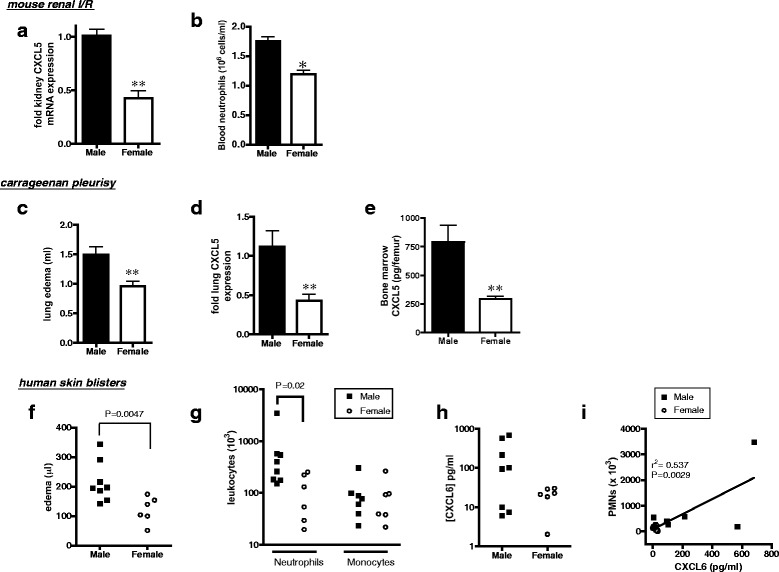
Increased induction of Cxcl5 in males is a common feature of I/R and other acute inflammatory responses. **a**–**d** Male and female C57BL6 mice were subjected to bilateral renal ischemia for 30 min followed by 24-h reperfusion (*n* = 4 mice/group). **a** Renal tissue Cxcl5 mRNA and **b** circulating GR1^hi^ neutrophils. **c**–**e** Carrageenan pleurisy was induced in male and female Wistar rats (0.15 ml of 1 % *w*/*v*, 3 h, *n* = 8 rats/group). **c** Excess volume of fluid recovered from pleural cavity (lung edema), **d** lung tissue Cxcl5 mRNA, **e** bone marrow Cxcl5 protein. Levels of mRNA are normalized to 18S and calculated as fold expression relative to mean value in males. Data are presented as mean ± sem. *P < 0.05 and **P < 0.01 by two-tailed unpaired Student’s *t* test. **f**–**i** Cantharidin-induced skin blisters in healthy male and female volunteers. **f** Blister volume (edema), **g** blister neutrophils and monocytes, measured by cytometry, **h** blister fluid CXCL6 protein, and **i** correlation between blister CXCL6 and neutrophils. Individual data points represent one volunteer. Blister samples were analyzed by non-parametric Mann-Whitney test

Renal dysfunction and neutrophil accumulation following I/R is more severe in males than that in females [10, 25]. Therefore, to understand whether sex-specific regulation of Cxcl5 in I/R occurs in other organs and mammals, we measured tissue and circulating chemokines following acute bilateral renal I/R in male and female mice. Renal ischemia of 30 min followed by 24-h reperfusion resulted in greater levels of tissue Cxcl5 mRNA in males than females (Fig. 5a) as well as plasma Cxcl5 protein (1070 ± 87 pg/ml and 540 ± 91, *P* < 0.01, *n* = 4, respectively). Similar to mesenteric I/R, this chemokine profile in males was also associated with more circulating neutrophils than that in females (Fig. 5b). Next, we examined whether disparate induction of Cxcl5 also dictates sex differences in neutrophil responses induced by an exogenous stimulus. Injection of carrageenan into the pleural cavity of rats results in greater accumulation of fluid (Fig. 5c) and neutrophils in the cavity in males than in females [8]. This sex difference in pleurisy was also associated with higher levels of Cxcl5 in the lung tissue and bone marrow of males compared to females (Fig. 5d,e).

To examine the role of Cxcl5 in the magnitude of neutrophil responses in humans, we used the cantharidin skin blister model of leukocyte trafficking in healthy volunteers. The mean age of participants was 21 ± 0.7 (males, *n* = 8) and 25 ± 1.6 (females, *n* = 6, *P* > 0.05). At 24 h following application of cantharidin, edema (blister volume) was substantially greater in men than women (Fig. 5f). Similar to responses in rodents, the number of neutrophils (but not monocytes) recruited to the site of injury was also greater in men compared to that in women (Fig. 5g). Levels of CXCL6, the human ortholog of rodent Cxcl5, tended to be higher in the blisters of men than women and were correlated with the number of neutrophils recovered from the blister (Fig. 5h,i). Thus, together, our findings indicate that increased induction of Cxcl5/CXCL6 in males in the early phases of innate immune responses is a common response to diverse pro-inflammatory stimuli in male mammals that contributes to elevated neutrophil stimulation and tissue infiltration.

## Discussion

Regulation of neutrophil accumulation in tissues is a critical feature of acute inflammation. Here, we provide novel insight into the nature and molecular mechanisms that determine sexual dimorphic regulation of neutrophil responses in I/R. Specifically, we provide evidence that Cxcl5/6 is the key molecular signal whose greater induction in males primarily affects circulating neutrophil numbers and adhesion molecule expression, thereby enhancing tissue infiltration.

Tissue injury during I/R involves multiple complex interacting mechanisms and several cell types (for review, see [26]). In this study, we focused on understanding sex differences in the initial inflammatory response during I/R and the early stages of neutrophil recruitment. We, and others, have demonstrated increased total neutrophil infiltration of tissues in males compared to females in acute inflammatory states [8–10]. In agreement with these studies, we observed substantially greater neutrophil influx into the peritoneal cavity following mesenteric I/R in male rats compared to age-matched females. Migration of blood neutrophils to sites of inflammatory insult is a multi-step process involving leukocyte rolling, adhesion, and emigration that is regulated by a network of chemokines, particularly ELR+ chemokines. In the current study, we aimed to understand the primary differences in neutrophil dynamics between males and females. Using IVM to monitor changes in I/R-induced leukocyte responses within the same animal in the mesenteric vasculature in real time, we reveal that the initial (<30 min) increase in leukocyte flux during reperfusion is similar between the sexes. In males, these responses continue to increase with reperfusion time, leading to accumulation of substantial number of neutrophils within the peritoneal cavity by 2 h. In contrast, the extent of leukocyte/vessel wall interactions in females remains relatively constant between 30-min and 2-h reperfusion. However, the velocity of rolling male leukocytes is similar to female cells, suggesting little differences in leukocyte motility and their ability to interact with blood vessels. Sex differences in neutrophil dynamics appear to be predominantly due to the greater neutrophilia that is evident in males throughout reperfusion. Therefore, the male neutrophil response does not appear to be an earlier onset or slower clearance of recruited cells compared to females but rather a difference in the absolute magnitude of the response brought about, in part, by more neutrophils available in the circulation at later stages of reperfusion. Indeed, the extent of tissue injury in the intestine is directly correlated with the number of circulating neutrophils and is therefore substantially greater in males. Similarly, several lines of evidence indicate that severity of inflammatory diseases are correlated with the extent of circulating neutrophils [27–29], pathologies that are also considered to be more severe in age-matched men compared to women. We believe that distinct regulation of neutrophil trafficking in males may be a fundamental feature of acute inflammatory responses.

Circulating neutrophil populations are rapidly increased in inflammatory responses, predominantly via elevated rates of trafficking out of bone marrow stores. Our study provides the first evidence for differential regulation of neutrophil pro-mobilization pathways in males and females. GCSF is a major mobilizing cytokine that directly regulates granulopoiesis and bone marrow neutrophil shape change, motility, and release [30] under homeostatic [31] and inflammatory conditions [32, 33]. Mesenteric I/R induces greater GCSF expression at the site of injury and within bone marrow cells, neutrophil shape change, and an increase in neutrophil integrin expression in males than in females. These findings are consistent with increased rates of trafficking of stimulated neutrophils into the circulation during reperfusion in males but not females.

Trafficking of neutrophils is predominantly guided by chemokines. In particular, archetypal ELR+ chemokines generated at the site of inflammation are not only direct neutrophil chemoattractants but also induce neutrophil mobilization from bone marrow stores, primarily through interaction with leukocyte receptor Cxcr2. For example, ELR+ chemokines Cxcl1 and Cxcl2 are required for bone marrow neutrophil motility and mobilization following administration of GCSF [30] or thioglycollate [24] in mice. In the current study, we identified Cxcl5 as the predominant ELR+ chemokine released throughout I/R in males but not females. Circulating and bone marrow levels of Cxcl5 are upregulated by I/R and increase with reperfusion time in males, with little induction of Cxcl5 evident in females. Indeed, blockade of Cxcl5 in males results in an identical neutrophil response as that seen in females. As such, leukocyte/vessel wall interactions within 45-min reperfusion are unaffected by Cxcl5 inhibition but leukocyte recruitment and tissue injury at later stages of reperfusion are substantially reduced. Thus, Cxcl5 has a predominant role in driving sustained neutrophil recruitment during I/R. At early time points, neutrophil infiltration is most likely mediated by other chemokines such as Cxcl1 and Ccl2 that are transiently induced in tissues of both sexes and can recruit neutrophils already in the circulation. The functional effect of Cxcl5 production during I/R in males is partly to increase circulating neutrophils since inhibition of Cxcl5 also reduces I/R-induced neutrophilia to similar levels seen in females. Conversely, administration of Cxcl5 to males in the absence of ischemic insult is sufficient to account for sex differences in I/R-induced changes in circulating neutrophil populations. Therefore, Cxcl5 has a non-redundant role in release of neutrophils into the circulation during I/R, which may be a general phenomenon during acute resolving/self-limiting inflammation.

Previous studies indicate an important homeostatic role of Cxcl5 in maintaining bone marrow neutrophil numbers [34]. However, our study shows no apparent sex difference in homeostatic functions of Cxcl5 since the levels and cell populations are similar in untreated male and female controls (data not shown) and in sham-operated animals. We only observe disparities in the functional role of Cxcl5 in males and females in response to pro-inflammatory stimuli where a temporal relationship between production of Cxcl5 and extent of neutrophil infiltration into tissue intimates a causal relation. Cxcl5 is an upstream regulator of GCSF production within bone marrow *in vivo* [35]. Similarly, in the current study, application of Cxcl5 to isolated bone marrow cells *in vitro* induced substantial expression of GCSF and neutrophil β2 integrin. Surprisingly, these effects on cell surface β2 integrin were greater in male than those in female cells despite similar basal levels of receptor Cxcr2 expression as confirmed by greater capacity of male cells to adhere to purified integrin ligand ICAM-1. Thus, male bone marrow cells have increased sensitivity to Cxcl5 through disparate Cxcr2 signaling; the precise mechanism is not elucidated in the current study but merits further investigation. Nonetheless, this important finding indicates that even in situations where Cxcl5 levels are elevated in females, circulating neutrophils are likely to have a less adhesive phenotype compared to neutrophils in males. The effect of Cxcl5 is clearly more potent than Cxcl1 in either sex, in agreement with previous reports indicating that truncated forms of Cxcl5 have greater affinity for CXCR2 than other ligands [36]. Administration of Cxcl5 also induces its own expression as well as its receptor, which suggests that Cxcl5 appears to work in a positive feedback loop to enhance and prolong synthesis at a transcriptional level. This may underlie differential kinetics of this chemokine whereby Cxcl5 has a longer half-life than other ELR+ chemokines. For example in acute endotoxemia in mice, Cxcl5 expression remains elevated longer than Cxcl1 and Cxcl2 in almost all tissues [37]. Moreover in severe inflammation, Cxcl5 production prolongs the activity of Cxcl1 and Cxcl2 by establishing local chemokine gradients and thereby promoting tissue injury [34].

Previous reports have demonstrated greater renal injury, neutrophil accumulation, and mortality rates in males than in females following renal I/R in rats [10]. Therefore, to assess whether sex-specific regulation of Cxcl5-mediated neutrophil responses occurs during I/R in other organs and species, we investigated sex differences in renal I/R in mice. Similar to our findings in rat mesenteric I/R, greater Cxcl5 expression was evident in the kidneys of male mice compared to females at the end of reperfusion and associated with elevated numbers of circulating neutrophils with higher β2 integrin expression. It is important to note that the induction of ischemia itself may be different in females since sex hormones, in particular estrogens, can limit the production of tissue-damaging mitochondrial reactive oxygen species and thereby increase tolerance to ischemia and reduce the inflammatory response (for review, see [38]). However, we observed the same divergent regulation of Cxcl5-dependent neutrophil responses in males and females following administration of equivalent amount of a chemical stimulus (carrageenan) into the pleural cavity of rats. Increased expression of Cxcl5 in the lung tissue and bone marrow of males was also associated with greater lung edema. Overall, our data imply that distinct regulation of Cxcl5 induction in males is a general phenomenon during I/R and other acute inflammatory responses that result in enhanced neutrophil accumulation and tissue injury.

In humans, Cxcl5 exists in two forms, namely CXCL5/ENA78 and CXCL6/GCP2. Recent evidence indicates that CXCL6/GCP2 is the closer ortholog to rodent Cxcl5 [15]. Our study in human volunteers supports the thesis that sex-specific regulation of CXCL6 underlies increased neutrophil recruitment in males. Topical application of cantharidin in healthy volunteers disrupts intercellular connections between keratinocytes and stimulates the influx of leukocytes, predominantly neutrophils, within 24 h [39]. While there is variation in the magnitude of leukocyte recruitment into blisters between individuals, the extent of neutrophil (but not monocyte) influx and edema is greater in men. Despite the small sample size, in agreement with our studies in rodents, local amounts of CXCL6 within the blister fluid are correlated with neutrophil accumulation and tend to be greater in men. Therefore, together, our findings indicate that reduced production and sensitivity to Cxcl5/6 may underlie a fundamental tissue-sparing mechanism in acute inflammatory responses in women. Furthermore, Cxcl5/6 may be an important therapeutic target in perturbing acute inflammatory diseases as well as a biochemical marker of disease progression.

Sex differences in regulation of CXCL6 may have important consequences for progression of acute inflammatory disorders. Few studies of human disease have investigated the biological role of CXCL6; however, ortholog CXCL5/ENA78 is involved in a variety of inflammatory diseases such as ARDS [40, 41], acute coronary syndromes, inflammatory bowel disease, asthma, and psoriasis, diseases known to have a neutrophil component. Serum levels of chemokine CXCL5 have also been associated with intima-media thickness of the common carotid artery, a marker of preclinical atherosclerosis, and CXCL5 levels are reduced by anti-inflammatory statin therapy [42]. Whether CXCL5 activity underlies sex differences in acute inflammatory disorders is not known. However, polymorphisms in CXCL5 gene are correlated with acute coronary syndrome [43], a disease where more numerous plaque-destabilizing neutrophils have been observed in lesions of men compared to women. We suggest that our findings indicate a role for increased CXCL5/6 induction in males in contributing to sex differences in inflammatory disorders that are characterized by excessive neutrophil recruitment.

Further investigations will be required to elucidate the upstream mechanisms that lead to greater induction of Cxcl5/6 in males compared to females. Our study does not distinguish whether male factors increase Cxcl5/6 or whether female factors inhibit Cxcl5/6. Current evidence indicates complex and interacting influences of sex on inflammatory responses including multiple sex hormones, X-linked and Y-linked genes (for reviews, see [3, 44, 45]), although no unifying explanation exists since these factors may paradoxically be pro- or anti-inflammatory depending on cell type, stimulus, stage of response, and age. Thus, to fully understand how sex differences in neutrophil responses are brought about, future studies need to examine the impact of individual sex hormones and chromosomes on regulation of chemokine production and neutrophil trafficking. Moreover, although our data demonstrate that the male neutrophil response can be made “female” by acutely blocking endogenous Cxcl5, it remains to be shown whether Cxcl5 alone is sufficient to make a female neutrophil response identical to that in males. In the current study, we primarily focused on the role of chemokine production on sex differences in neutrophil dynamics in the onset phases of leukocyte recruitment. The data suggest that a primary component of the sex difference in neutrophil trafficking is due to a difference in the number of circulating neutrophils and Cxcl5-stimulated recruitment. However, several other components of the inflammatory response may also be differentially regulated between the sexes and contribute to greater neutrophil accumulation in male tissues. For example, it is possible that differences in leukocyte cytoskeleton integrity or function may affect leukocyte motility and impede the recruitment of female leukocytes into tissues or their release from the bone marrow due to differences in signaling of adhesion molecules such as PSGL-1 and L-selectin or a difference in the ability of neutrophils to deform and transmigrate. Previous studies also indicate that female sex hormones can influence the life span of neutrophils [46], a finding that may have implications for the number of neutrophils in the circulation and tissue, particularly in later stages of inflammation. Our data suggest greater Cxcl5/6 production in males in different inflammatory models in three species; however, further investigations with larger sample size will be needed to fully establish the existence and consequence of differential Cxcl5/6 production between the sexes in inflammatory disorders. Detailed understanding of fundamental differences in male and female neutrophil responses may lead to improved anti-inflammatory therapies.

## Conclusions

In conclusion, acute inflammatory insult initiates distinct neutrophil responses in males and females. A salient feature of this difference comprises an increased production and sensitivity to chemokine Cxcl5/6 in males. The data demonstrate a novel and predominant role for Cxcl5/6 in sustaining neutrophil recruitment into tissues partly through mobilization of tissue-damaging neutrophils from bone marrow. These findings may have important implications for understanding the basis of sex differences in neutrophil trafficking in diverse acute inflammatory pathologies.

## Additional files

Additional file 1:Tables S1 and S2.
**Table S1.** Details of antibodies used for flow cytometry. **Table S2.** Sequence of primers used for real-time quantitative PCR with SYBR green. (PDF 96 kb) Additional file 2: Figure S1. Distinct temporal regulation of neutrophil recruitment increases tissue I/R injury in males. Male and female rats were subjected to 30-min mesenteric ischemia followed by up to 2-h reperfusion. (A) Representative images of portions of male and female small intestine at the end of reperfusion, demonstrating redness and edema in males following I/R but no observable change in females. (B-C) Leukocyte/vessel wall interactions throughout reperfusion in mesenteric venules, measured by intravital microscopy: (B) Leukocyte adhesion and (C) number of tissue leukocytes at different distances away from venule (sham, *n* = 5 rats/group; I/R, *n* = 8 rats/group). (D) Circulating CD68+ monocytes in males and females during ischemia and reperfusion. Data are presented as mean ± sem. §*P* < 0.001 by two-way ANOVA and ‡*P* < 0.05 or #*P* < 0.001 by Bonferroni’s post-test. ns denotes *P* > 0.05 by one-way ANOVA. (PDF 811 kb) Additional file 3: Figure S2. No effect of sex or ischemia on hemodynamics during reperfusion. Male and female rats were subjected to 30-min mesenteric ischemia followed by up to 2-h reperfusion. (A) Mean arterial blood pressure (MABP) measured by cannulation of carotid artery. (B) Red blood cell (RBC) velocity and (C) wall shear rate, calculated from RBC velocity and venule diameter, as described in Supplementary Methods. Sham, *n* = 4 rats/group; I/R, *n* = 4 rats/group. Data are presented as mean ± sem. All comparisons *P* > 0.05 by two-way ANOVA. (TIFF 1174 kb) Additional file 4: Figure S3. Increased induction of neutrophil integrins in males. Male and female rats were subjected to 30-min mesenteric ischemia followed by 2-h reperfusion. Surface expression of integrins β1, α4, αL, and αM on RP1+ neutrophils in BM and circulation of male and female rats, measured at 2-h reperfusion. Sham, *n* = 3 rats/group; I/R, *n* = 5 rats/group. Data are presented as mean ± sem. **P* < 0.05, ***P* < 0.01 by one-way ANOVA followed by Bonferroni’s post-test. (TIFF 540 kb) Additional file 5: Figure S4. Sex-specific regulation of Cxcl5 determines magnitude of leukocyte dynamics in I/R. (A-D) Male and female rats were subjected to 30-min mesenteric ischemia followed by up to 2-h reperfusion. (A) Mesenteric tissue Ccl2 mRNA and (B) plasma protein Ccl2, at 30-min and 2-h reperfusion. (C-D) Mesenteric tissue Ccl3 and Ccl5 mRNA levels, at 30-min and 2-h reperfusion. Levels of mRNA are normalized to 18S and calculated as fold expression relative to mean value in sham-operated males. Sham, *n* = 5 rats/group; I/R, *n* = 8 rats/group. (E-F) Male rats were treated with either anti-Cxcl5 (20 μg/kg, iv) or control IgG (20 μg/kg, iv) 1 h prior to mesenteric ischemia (*n* = 5 rats/group). (E) Number of adherent leukocytes and (F) number of emigrated leukocytes within 50 μm of blood vessel wall, measured by intravital microscopy during reperfusion. Data are presented as mean ± sem. **P* < 0.05, by one-way ANOVA followed by Bonferroni’s post-test. §*P* < 0.001 by two-way ANOVA and ‡*P* < 0.05, ***P* < 0.01 or #*P* < 0.001 by Bonferroni’s post-test. (TIFF 2343 kb) Additional file 6: Figure S5. Cxcl5-induced leukocyte/vessel wall interactions. Male rats were treated with 3 μg/kg Cxcl5 (ip, *n* = 5 rats) or PBS (*n* = 3 rats) for 2 h. (A) Leukocyte flux, (B) adherent leukocytes, (C) emigrated leukocytes in mesenteric tissues at 2 h. Data are presented as mean ± sem. **P* < 0.05,***P* < 0.01, ****P* < 0.001 compared to PBS by Student’s *t* test. (TIFF 448 kb)

## References

[1] Nathan C (2002). Points of control in inflammation. Nature.

[2] Whitacre CC (2001). Sex differences in autoimmune disease. Nature Immunol.

[3] Casimir GJ, Lefèvre N, Corazza F, Duchateau J (2013). Sex and inflammation in respiratory diseases: a clinical viewpoint. Biol Sex Differ.

[4] Sperry JL, Minei JP (2008). Gender dimorphism following injury: making the connection from bench to bedside. J Leuk Biol.

[5] Murphy E, Steenbergen C (2007). Gender-based differences in mechanisms of protection in myocardial ischemia-reperfusion injury. Cardiovasc Res.

[6] Moss M, Mannino DM (2002). Race and gender differences in acute respiratory distress syndrome deaths in the United States: an analysis of multiple-cause mortality data (1979- 1996). Crit Care Med.

[7] Kim AM, Tingen CM, Woodruff TK (2010). Sex bias in trials and treatment must end. Nature.

[8] Scotland RS, Stables MJ, Madalli S, Watson P, Gilroy DW (2011). Sex-differences in resident immune cell phenotype underlies more efficient acute inflammatory responses in female mice. Blood.

[9] Villar IC, Scotland RS, Khambata RS, Chan M, Duchene J, Sampaio AL (2011). Suppression of endothelial P-Selectin expression contributes to reduced cell trafficking in females: an effect independent of NO and prostacyclin. Arterioscl Thr Vasc Biol.

[10] Robert R, Ghazali DA, Favreau F, Mauco G, Hauet T, Goujon J-M (2011). Gender difference and sex hormone production in rodent renal ischemia reperfusion injury and repair. J Inflamm.

[11] Zlotnik A, Yoshie O (2000). Chemokines: a new classification system and their role in immunity. Immunity.

[12] McDonald B, Pittman K, Menezes GB, Hirota SA, Slaba I, Waterhouse CC (2010). Intravascular danger signals guide neutrophils to sites of sterile inflammation. Science.

[13] Nourshargh S, Hordijk PL, Sixt M (2010). Breaching multiple barriers: leukocyte motility through venular walls and the interstitium. Nat Rev Mol Cell Biol.

[14] Summers C, Rankin SM, Condliffe AM, Singh N, Peters AM, Chilvers ER (2010). Neutrophil kinetics in health and disease. Trends Immunol.

[15] Nomiyama H, Osada N, Yoshie O (2010). The evolution of mammalian chemokine genes. Cytokine Growth Factor Rev.

[16] Nourshargh S, Larkin SW, Das A, Williams TJ (1995). Interleukin-1-induced leukocyte extravasation across rat mesenteric microvessels is mediated by platelet-activating factor. Blood.

[17] Cole AT, Garlick NM, Galvin AM, Hawkey CJ, Robins RA (1995). A flow cytometric method to measure shape change of human neutrophils. Clin Sci.

[18] Macey MG, McCarthy DA, Howells GL, Curtis MA, King G, Newland AC (1998). Multiparameter flow cytometric analysis of polymorphonuclear leucocytes in whole blood from patients with adult rapidly progressive periodontitis reveals low expression of the adhesion molecule L-selectin (Cd62L). Cytometry.

[19] Livak KJ, Schmittgen TD (2001). Analysis of relative gene expression data using real-time quantitative PCR and the 2 ^-ΔΔCT^ method. Methods.

[20] Jenner W, Motwani M, Veighey K, Newson J, Audzevich T, Nicolaou A (2014). Characterisation of leukocytes in a human skin blister model of acute inflammation and resolution. PLoS One.

[21] Powell RJ, Cronenwett JL, Gauthier AJ, Wagner RJ (1995). Quantitating intestinal ischemia with nitroblue tetrazolium salts. J Surgical Res.

[22] Panopoulos AD, Watowich SS (2008). Granulocyte colony-stimulating factor: molecular mechanisms of action during steady state and ‘emergency’ hematopoiesis. Cytokine.

[23] Carden DL, Granger DN (2000). Pathophysiology of ischemia-reperfusion injury. J Pathol.

[24] Wengner AM, Pitchford SC, Furze RC, Rankin SM (2008). The coordinated action of G-CSF and ELR + CXC chemokines in neutrophil mobilization during acute inflammation. Blood.

[25] Kher A, Meldrum KK, Wang M, Tsai BM, Pitcher JM, Meldrum DR (2005). Cellular and molecular mechanisms of sex differences in renal ischemia-reperfusion injury. Cardiovasc Res.

[26] Kalogeris T, Baines CP, Korthuis RJ (2012). Cell biology of ischemia/reperfusion injury. Int Rev Cell Mol Biol.

[27] Kreisel D, Sugimoto S, Tietjens J, Zhu J, Yamamoto S, Krupnick AS (2011). Bcl3 prevents acute inflammatory lung injury in mice by restraining emergency granulopoiesis. J Clin Invest.

[28] Gomes AL, Carvalho T, Serpa J, Torre C, Dias S (2010). Hypercholesterolemia promotes bone marrow cell mobilization by perturbing the SDF-1:CXCR4 axis. Blood.

[29] Delano MJ, Kelly-Scumpia KM, Thayer TC, Winfield RD, Scumpia PO, Cuenca AG (2011). Neutrophil mobilization from the bone marrow during polymicrobial sepsis is dependent on CXCL12 signaling. J Immunol.

[30] Köhler A, De Filippo K, Hasenberg M, van den Brandt C, Nye E, Hosking MP (2011). G-CSF-mediated thrombopoietin release triggers neutrophil motility and mobilization from bone marrow via induction of Cxcr2 ligands. Blood.

[31] Forlow SB, Schurr JR, Kolls JK, Bagby GJ, Schwarzenberger PO, Ley K (2001). Increased granulopoiesis through interleukin-17 and granulocyte colony-stimulating factor in leukocyte adhesion molecule-deficient mice. Blood.

[32] Kim HK, De La Luz SM, Williams CK, Gulino AV, Tosato G (2006). G-CSF down-regulation of CXCR4 expression identified as a mechanism for mobilization of myeloid cells. Blood.

[33] Stark MA, Huo Y, Burcin TL, Morris MA, Olson TS, Ley K (2005). Phagocytosis of apoptotic neutrophils regulates granulopoiesis via IL-23 and IL-17. Immunity.

[34] Mei J, Liu Y, Dai N, Favara M, Greene T, Jeyaseelan S (2010). CXCL5 regulates chemokine scavenging and pulmonary host defense to bacterial infection. Immunity.

[35] Mei J, Liu Y, Dai N, Hoffmann C, Hudock KM, Zhang P (2012). Cxcr2 and Cxcl5 regulate the IL-17/G-CSF axis and neutrophil homeostasis in mice. J Clin Invest.

[36] Mortier A, Loos T, Gouwy M, Ronsse I, Van Damme J, Proost P (2010). Posttranslational modification of the NH2-terminal region of CXCL5 by proteases or peptidylarginine Deiminases (PAD) differently affects its biological activity. J Biol Chem.

[37] Rovai LE, Herschman HR, Smith JB (1998). The murine neutrophil-chemoattractant chemokines LIX, KC, and MIP-2 have distinct induction kinetics, tissue distributions, and tissue-specific sensitivities to glucocorticoid regulation in endotoxemia. J Leuk Biol.

[38] Xing D, Nozell S, Oparil S (2009). Estrogen and mechanisms of vascular protection. Arterioscl Thromb Vasc Biol.

[39] Day RM, Harbord M, Forbes A, Segal AW (2001). Cantharidin blisters: a technique for investigating leukocyte trafficiking and cytokine production at sites of inflammation in humans. J Immunol Methods.

[40] Goodman RB, Strieter RM, Martin DP, Steinberg KP, Milberg JA, Maunder RJ (1996). Inflammatory cytokines in patients with persistence of the acute respiratory distress syndrome. Am J Resp Crit Care Med.

[41] Imaizumi T, Albertine KH, Jicha DL, McIntyre TM, Prescott SM, Zimmerman GA (1997). Human endothelial cells synthesize ENA-78: relationship to IL-8 and to signaling of PMN adhesion. Am J Resp Cell Mol Biol.

[42] Chen L, Yang Z, Lu B, Li Q, Ye Z, He M (2011). Serum CXC ligand 5 is a new marker of subclinical atherosclerosis in type 2 diabetes. Clin Endocrinol.

[43] Zineh I, Beitelshees AL, Welder GJ, Hou W, Chegini N, Wu J (2008). Epithelial neutrophil-activating peptide (ENA-78), acute coronary syndrome prognosis, and modulatory effect of statins. PLoS One.

[44] Straub RH (2007). The complex role of estrogens in inflammation. Endocr Rev.

[45] Case LK, Teuscher C (2015). Y genetic variation and phenotypic diversity in health and disease. Biol Sex Diff.

[46] Molloy EJ, O’Neill AJ, Grantham JJ, Sheridan-Pereira M, Fitzpatrick JM, Webb DW (2003). Sex-specific alterations in neutrophil apoptosis: the role of estradiol and progesterone. Blood.

