# Supplementary material for: Sex-specific regulation of chemokine Cxcl5/6 controls neutrophil recruitment and tissue injury in acute inflammatory states
Source: Biol Sex Differ. 2015 Nov 26;6:27. doi: 10.1186/s13293-015-0047-5 (PMC4661984; doi:10.1186/s13293-015-0047-5)
Supplement: Additional file 1:Tables S1 and S2. — Table S1. Details of antibodies used for flow cytometry. Table S2. Sequence of primers used for real-time quantitative PCR with SYBR green. (PDF 96 kb) [file 13293_2015_47_MOESM1_ESM.pdf]

**Supplemental Information**
Supplemental information includes 5 figures and 2 tables.

**Supplementary Figure Legends**

**Figure S1. Distinct temporal regulation of neutrophil recruitment increases tissue I/R injury in males.** Male and female rats were subjected to 30min mesenteric ischemia followed by up to 2h reperfusion. (A) Representative images of portions of male and female small intestine at the end of reperfusion, demonstrating redness and edema in males following I/R but no observable change in females. (B-C) Leukocyte/vessel wall interactions throughout reperfusion in mesenteric venules, measured by intravital microscopy: (B) Leukocyte adhesion and (C) number of tissue leukocytes at different distances away from venule (sham: n=5 rats/group, I/R: n=8 rats/group). (D) Circulating CD68+ monocytes in males and females during ischemia and reperfusion. Data are presented as mean ± sem. §P<0.001 by two-way ANOVA, and ‡P<0.05 or #P<0.001 by Bonferroni's post-test. ns denotes P>0.05 by one-way ANOVA.

**Figure S2. No effect of sex or ischemia on hemodynamics during reperfusion.** Male and female rats were subjected to 30min mesenteric ischemia followed by up to 2h reperfusion. (A) Mean arterial blood pressure (MABP) measured by cannulation of carotid artery. (B) Red blood cell (RBC) velocity and (C) wall shear rate, calculated from RBC velocity and venule diameter, as described in Supplementary Methods. Sham: n=4 rats/group, I/R: n=4 rats/group. Data are presented as mean ± sem. All comparisons P>0.05 by two-way ANOVA.

**Figure S3. Increased induction of neutrophil integrins in males.** Male and female rats were subjected to 30min mesenteric ischemia followed by 2h reperfusion. Surface expression of integrins $\beta1$, $\alpha4$, $\alpha L$ and $\alpha M$ on RP1+ neutrophils in BM and circulation of male and female rats, measured at 2h reperfusion. Sham: n=3 rats/group, I/R: n=5 rats/group. Data are presented as mean ± sem. *P<0.05, **P<0.01 by one-way ANOVA followed by Bonferroni's post-test.

**Figure S4. Sex-specific regulation of Cxcl5 determines magnitude of leukocyte dynamics in I/R.** (A-D) Male and female rats were subjected to 30min mesenteric ischemia followed by up to 2h reperfusion. (A) Mesenteric tissue Ccl2 mRNA and (B) plasma protein Ccl2, at 30min and 2h reperfusion. (C-D) Mesenteric tissue Ccl3 and Ccl5 mRNA levels, at 30min and 2h reperfusion. Levels of mRNA are normalized to 18S and calculated as fold expression relative to mean value in sham-operated males. Sham: n=5 rats/group, I/R: n=8 rats/group. (E-F) Male rats were treated with either anti-Cxcl5 (20µg/kg, iv) or control IgG (20µg/kg, iv) 1h prior to mesenteric ischemia (n=5 rats/group). (E) Number of adherent leukocytes and (F) number of emigrated leukocytes within 50µm of blood vessel wall, measured by intravital microscopy during reperfusion. Data are presented as mean ± sem. *P<0.05, by one-way ANOVA followed by Bonferroni's post-test. § P<0.001 by two-way ANOVA and ‡P<0.05, **P<0.01 or #P<0.001 by Bonferroni's post-test.

**Figure S5. Cxcl5-induced leukocyte/vessel wall interactions.** Male rats were treated with 3µg/kg Cxcl5 (ip, n=5 rats) or PBS (n=3 rats) for 2h. (A) Leukocyte flux, (B) adherent leukocytes, (C) emigrated leukocytes in mesenteric tissues at 2h. Data are presented as mean ± sem. *P<0.05,**P<0.01, ***P<0.001 compared to PBS by Student's t-test.

**Table S1. Details of antibodies used for flow cytometry.**

| Antigen | Clone | Supplier |
|---|---|---|
| ***Rat*** | | |
| Granulocytes | RP1 | BDPharmingen |
| CD68 | ED1 | AbD serotec |
| Integrin β1/CD29 | HMβ1-1 | Biolegend |
| Integrin β2/CD18 | WT.3 | AbD serotec |
| Integrin αL/CD11a | WT.1 | AbD serotec |
| Integrin αM/CD11b | OX-42 | AbD serotec |
| Integrin α4/CD49d | MRα4-1 | Biolegend |
| L-selectin/CD62L | OX-85 | Biolegend |
| | | |
| ***Mouse*** | | |
| GR1 | RB6-8C5 | BDPharmingen |
| Integrin β2/CD18 | C71/16 | BDPharmingen |
| | | |
| ***Human*** | | |
| CD14 | M5E2 | BDPharmingen |
| CD16 | 3G8 | BDPharmingen |
| CD3 | UCHT1 | BDPharmingen |
| HLA-DR | G46-6 | BD Horizon |

**Table S2. Sequence of primers used for real-time quantitative PCR with SYBR green.**

| | Forward | Reverse |
|---|---|---|
| ***RAT*** | | |
| **CXCL1/KC/GROα** | GGCAGGGATTCACTTCAAGA | GCCATCGGTGCAATCTATCT |
| **CXCL5/LIX** | CTCAAGCTGCTCCTTTCTCG | GCGATCATTTTGGGGTTAAT |
| **CCL2/MCP1** | ATGCAGTTAATGCCCCACTC | TTCCTTATTGGGGTCAGCAC |
| **CCL3/MIP1α** | TTTTGAGACCAGCAGCCTTT | CTCAAGCCCCTGCTCTACAC |
| **CCL5/RANTES** | GTGCCCACGTGAAGGAGTAT | ATCCCCAGCTGGTTAGGACT |
| **GCSF** | CCTAGCAGGCATTTCCTCTG | GCTGGAAGGCAGAAGTGAAG |
| **CXCR2** | CCAAGCTGATCAAGGAGACC | GGGGTTAAGACAGCTGTGGA |
| **18S** | AGCCTGCGGCTTAATTTGAC | CAACCTAAGACGGCCATGCA |
| | | |
| ***MOUSE*** | | |
| **CXCL5/LIX** | GCATTTCTGTTGCTGTTCACGCT | CCTCCTTCTGGTTTTTCAGTTTAGC |
