# Supplementary figures and images for: Sex-specific regulation of chemokine Cxcl5/6 controls neutrophil recruitment and tissue injury in acute inflammatory states
Source: Biol Sex Differ. 2015 Nov 26;6:27. doi: 10.1186/s13293-015-0047-5 (PMC4661984; doi:10.1186/s13293-015-0047-5)

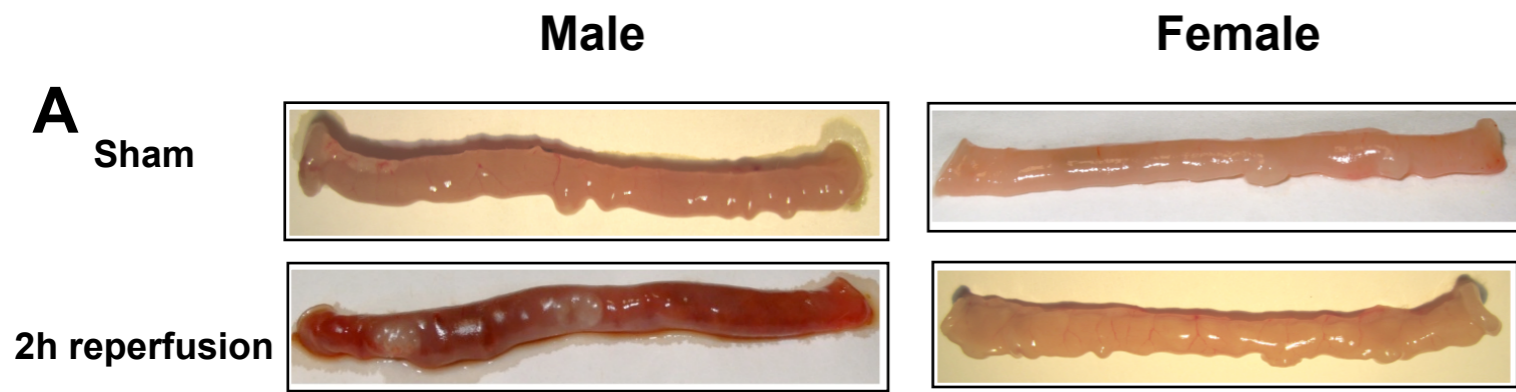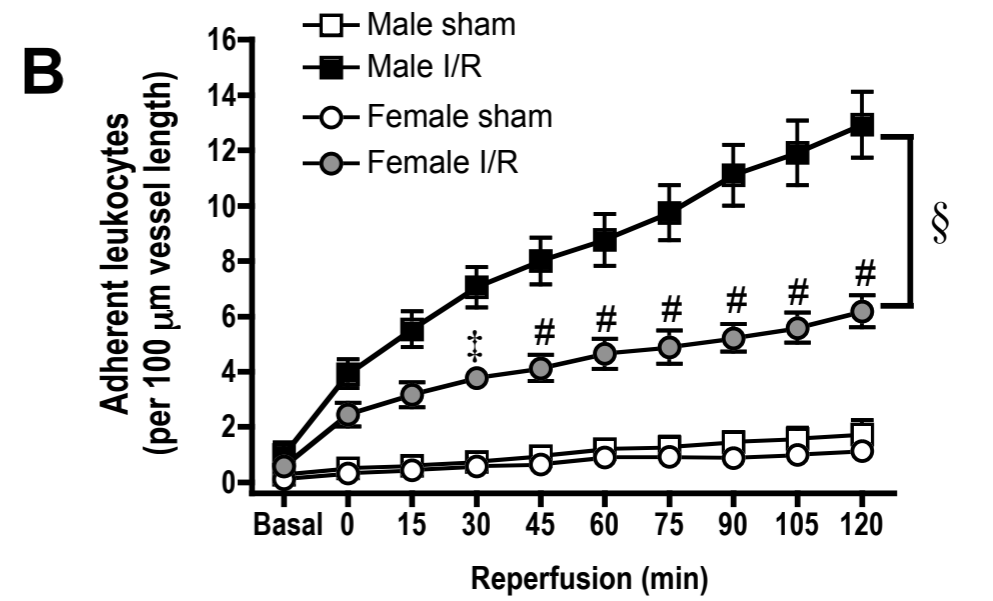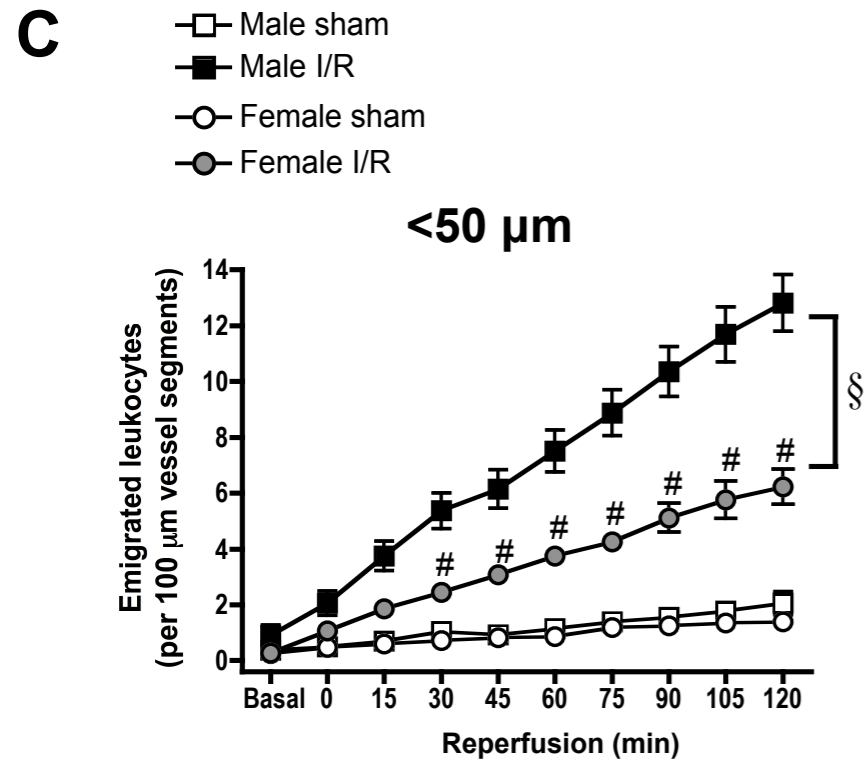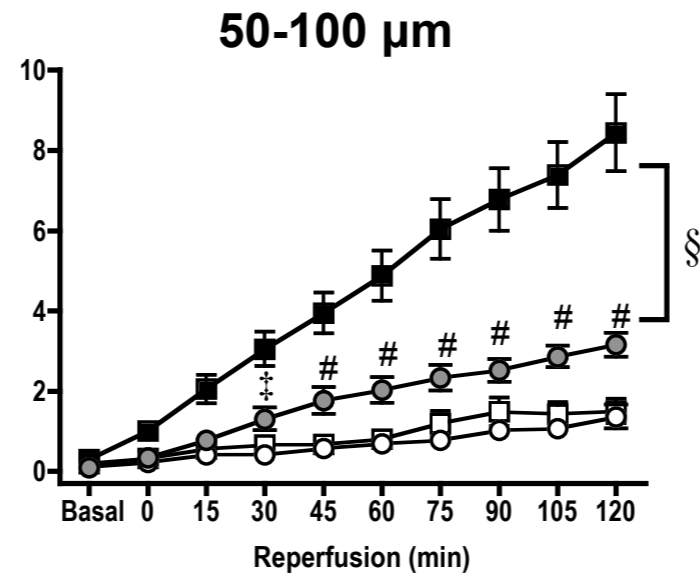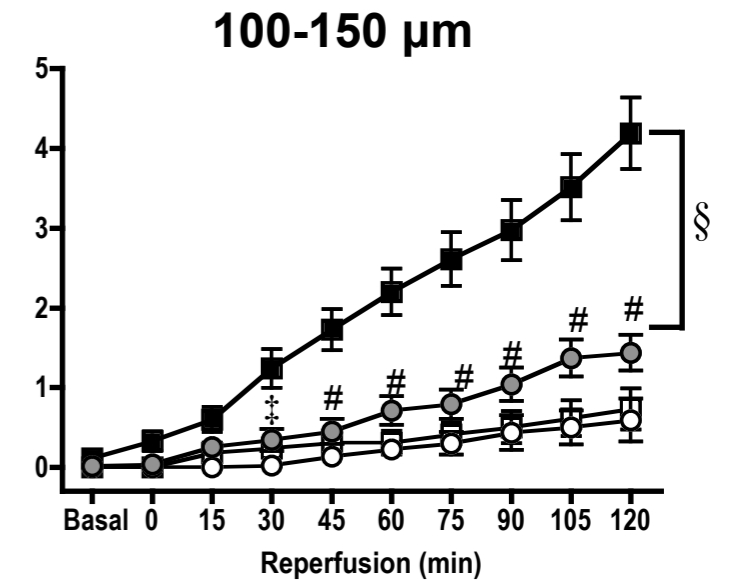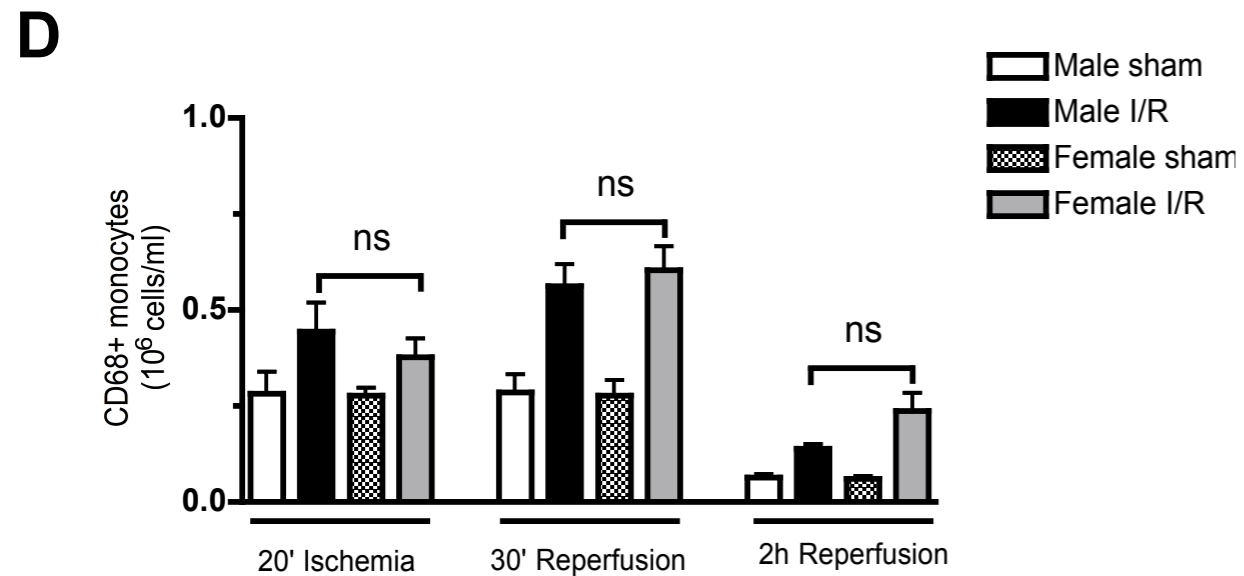

Supplement: Additional file 2: Figure S1. — Distinct temporal regulation of neutrophil recruitment increases tissue I/R injury in males. Male and female rats were subjected to 30-min mesenteric ischemia followed by up to 2-h reperfusion. (A) Representative images of portions of male and female small intestine at the end of reperfusion, demonstrating redness and edema in males following I/R but no observable change in females. (B-C) Leukocyte/vessel wall interactions throughout reperfusion in mesenteric venules, measured by intravital microscopy: (B) Leukocyte adhesion and (C) number of tissue leukocytes at different distances away from venule (sham, n = 5 rats/group; I/R, n = 8 rats/group). (D) Circulating CD68+ monocytes in males and females during ischemia and reperfusion. Data are presented as mean ± sem. §P < 0.001 by two-way ANOVA and ‡P < 0.05 or #P < 0.001 by Bonferroni’s post-test. ns denotes P > 0.05 by one-way ANOVA. (PDF 811 kb) [file 13293_2015_47_MOESM2_ESM.pdf]

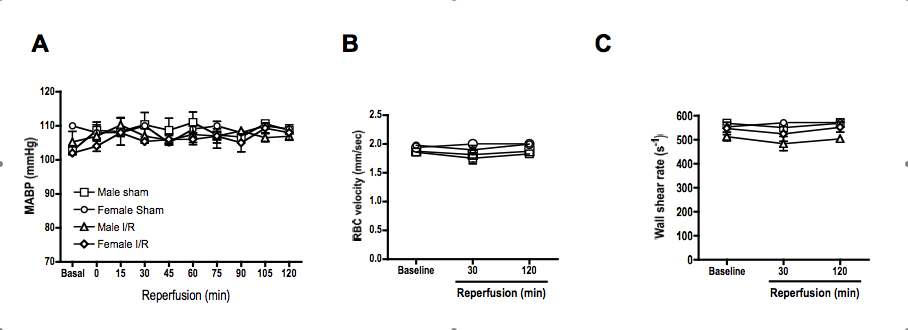

Supplement: Additional file 3: Figure S2. — No effect of sex or ischemia on hemodynamics during reperfusion. Male and female rats were subjected to 30-min mesenteric ischemia followed by up to 2-h reperfusion. (A) Mean arterial blood pressure (MABP) measured by cannulation of carotid artery. (B) Red blood cell (RBC) velocity and (C) wall shear rate, calculated from RBC velocity and venule diameter, as described in Supplementary Methods. Sham, n = 4 rats/group; I/R, n = 4 rats/group. Data are presented as mean ± sem. All comparisons P > 0.05 by two-way ANOVA. (TIFF 1174 kb) [file 13293_2015_47_MOESM3_ESM.tiff]

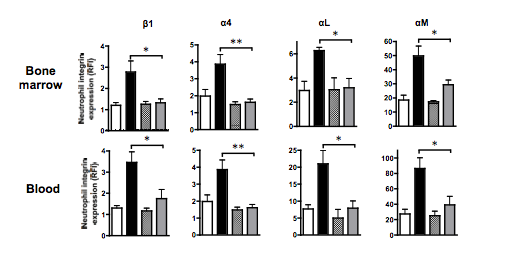

Supplement: Additional file 4: Figure S3. — Increased induction of neutrophil integrins in males. Male and female rats were subjected to 30-min mesenteric ischemia followed by 2-h reperfusion. Surface expression of integrins β1, α4, αL, and αM on RP1+ neutrophils in BM and circulation of male and female rats, measured at 2-h reperfusion. Sham, n = 3 rats/group; I/R, n = 5 rats/group. Data are presented as mean ± sem. *P < 0.05, **P < 0.01 by one-way ANOVA followed by Bonferroni’s post-test. (TIFF 540 kb) [file 13293_2015_47_MOESM4_ESM.tiff]

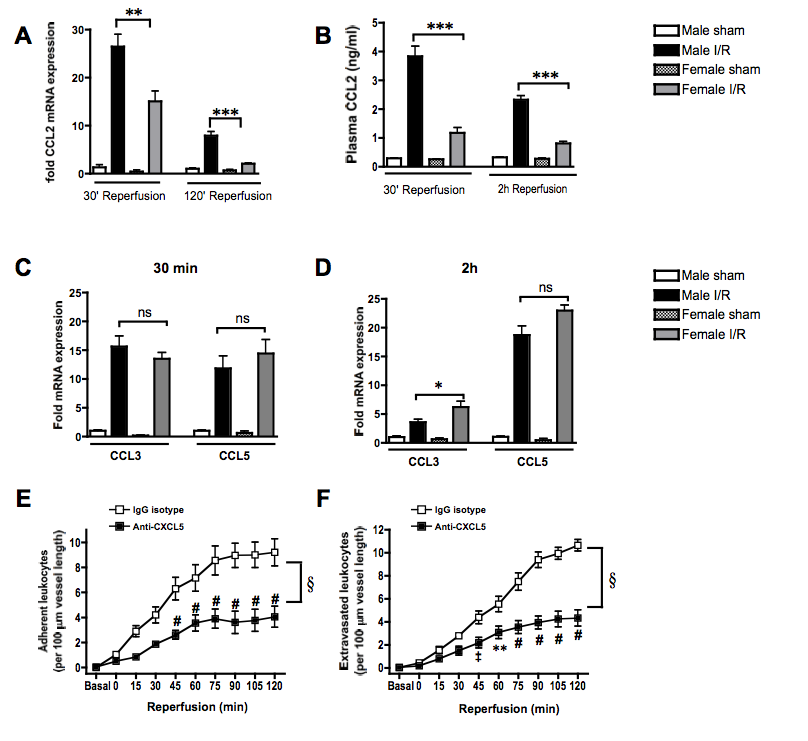

Supplement: Additional file 5: Figure S4. — Sex-specific regulation of Cxcl5 determines magnitude of leukocyte dynamics in I/R. (A-D) Male and female rats were subjected to 30-min mesenteric ischemia followed by up to 2-h reperfusion. (A) Mesenteric tissue Ccl2 mRNA and (B) plasma protein Ccl2, at 30-min and 2-h reperfusion. (C-D) Mesenteric tissue Ccl3 and Ccl5 mRNA levels, at 30-min and 2-h reperfusion. Levels of mRNA are normalized to 18S and calculated as fold expression relative to mean value in sham-operated males. Sham, n = 5 rats/group; I/R, n = 8 rats/group. (E-F) Male rats were treated with either anti-Cxcl5 (20 μg/kg, iv) or control IgG (20 μg/kg, iv) 1 h prior to mesenteric ischemia (n = 5 rats/group). (E) Number of adherent leukocytes and (F) number of emigrated leukocytes within 50 μm of blood vessel wall, measured by intravital microscopy during reperfusion. Data are presented as mean ± sem. *P < 0.05, by one-way ANOVA followed by Bonferroni’s post-test. §P < 0.001 by two-way ANOVA and ‡P < 0.05, **P < 0.01 or #P < 0.001 by Bonferroni’s post-test. (TIFF 2343 kb) [file 13293_2015_47_MOESM5_ESM.tiff]

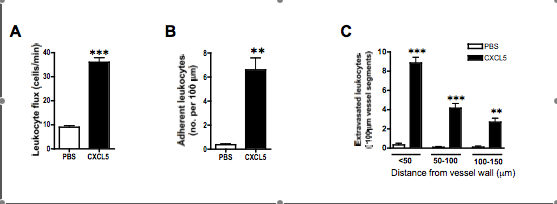

Supplement: Additional file 6: Figure S5. — Cxcl5-induced leukocyte/vessel wall interactions. Male rats were treated with 3 μg/kg Cxcl5 (ip, n = 5 rats) or PBS (n = 3 rats) for 2 h. (A) Leukocyte flux, (B) adherent leukocytes, (C) emigrated leukocytes in mesenteric tissues at 2 h. Data are presented as mean ± sem. *P < 0.05,**P < 0.01, ***P < 0.001 compared to PBS by Student’s t test. (TIFF 448 kb) [file 13293_2015_47_MOESM6_ESM.tiff]
